# Early Detection and Chemoprevention of Lung Cancer

**DOI:** 10.12688/f1000research.12433.1

**Published:** 2018-01-16

**Authors:** Melissa New, Robert Keith

**Affiliations:** 1 Division of Pulmonary Sciences and Critical Care Medicine, University of Colorado, Anschutz Medical Campus, Aurora, Colorado, USA; 2 VA Eastern Colorado Health Care System, Denver, Colorado, USA; 3 University of Colorado Denver, Aurora, Colorado, USA

**Keywords:** chemoprevention, cancer prevention, lung cancer screening

## Abstract

Despite advances in targeted treatments, lung cancer remains a common and deadly malignancy, in part owing to its typical late presentation. Recent developments in lung cancer screening and ongoing efforts aimed at early detection, treatment, and prevention are promising areas to impact the mortality from lung cancer. In the past several years, lung cancer screening with low-dose chest computed tomography (CT) was shown to have mortality benefit, and lung cancer screening programs have been implemented in some clinical settings. Biomarkers for screening, diagnosis, and monitoring of response to therapy are under development. Prevention efforts aimed at smoking cessation are as crucial as ever, and there have been encouraging findings in recent clinical trials of lung cancer chemoprevention. Here we review advancements in the field of lung cancer prevention and early malignancy and discuss future directions that we believe will result in a reduction in the mortality from lung cancer.

## Introduction

Lung cancer accounts for more cancer-related deaths than the next three deadliest cancers combined ^1^. Over the past decade, there have been advances in targeted treatments for lung cancer, but the survival from lung cancer has not dramatically improved, in large part because most patients present with advanced-stage disease that has already spread, making cure more difficult. In order to impact lung cancer mortality, efforts aimed at prevention and early detection are crucial. Here, we will review the literature and recent advances in early detection of lung cancer as well as lung cancer prevention efforts, including chemoprevention.

## Early detection: advances in lung cancer screening

The high mortality from lung cancer has inspired investigators for decades to search for effective lung cancer screening modalities. Early studies investigated chest X-ray and sputum cytology to identify higher-risk patients ^2^. Unfortunately, even though early screening efforts diagnosed more lung cancers, they did not translate to a decreased mortality from lung cancer, suggesting that lung cancers were still not being identified at an early enough stage to improve overall mortality ^3, 4^.

In 2011, the results of the National Lung Screening Trial (NLST) were published ^5^. This study evaluated only patients who were identified as high risk: those who were 55 to 74 years old, had a smoking history of at least 30 pack-years, and either were active smokers or had quit less than 15 years prior. The study randomly assigned over 53,000 patients to screening with either chest X-ray or low-dose chest computed tomography (CT) scan every year for three years and then followed them over time. There was a positive screen (showing a non-calcified nodule or mass at least 4 mm in any diameter) in 16% of the chest X-ray group and in 39% of the chest CT group. In follow-up of a positive screen result, there was further diagnostic testing, which typically involved only further imaging, although biopsies and other invasive procedures were performed when clinically relevant and lung cancers were diagnosed in 5.5% of abnormalities identified in the chest X-ray group and in 3.6% of those seen in the chest CT group. The study concluded that there was a 20% decrease in mortality from lung cancer in the CT group compared with chest X-ray after three rounds of annual screening over multiple years of follow-up. Put another way, 320 high-risk patients would need to be screened over three years to prevent one lung cancer death, and this compares favorably with other screening tests such as annual mammography for breast cancer screening (number needed to screen of more than 380 for women who are 60 to 69 years old and of more than 1,000 for those who are 50 to 59) and colon cancer screening with flexible sigmoidoscopy plus fecal occult blood test (number needed to screen of more than 350). The drawbacks include the fact that the majority of nodules detected were not malignant, and this leads to more imaging and to possible invasive diagnostic procedures, although the rates of harm from these were low in the NLST.

Following the publication of the NLST results, the US Preventive Services Task Force (USPSTF) recommended that annual lung cancer screening with a low-dose chest CT be implemented in the same high-risk group that was studied in the NLST, although they extended their recommendation to include patients up to age 80. We are now in the implementation phase of lung cancer screening and have begun to understand the challenges involved. Tobacco cessation is a crucial part of a lung cancer screening program, as this is currently the only intervention that can prevent the development of lung cancer. Shared decision-making between physicians and patients to initiate lung cancer screening on an individual basis is important, especially given that positive screens are relatively common (27% of initial CT scans in the NLST) and frequently require further monitoring or evaluation ^5^. In fact, the Centers for Medicare & Medicaid Services (CMS) requires shared decision-making to be documented as part of a visit for lung cancer screening ^6^. Online tools to assist with a shared decision-making conversation have provided helpful visual aids, such as www.shouldiscreen.com. Radiologists have also worked to standardize low-dose CT interpretation and developed recommendations for the follow-up of abnormalities identified through screening CTs: the Lung-Reporting and Data System (Lung-RADS) recommendations ^7^. When these guidelines were applied retrospectively to patients in the NLST, the positive predictive value of a positive screen improved ^8^.

Now that the USPSTF and CMS have endorsed lung cancer screening, various health-care systems have started to implement screening programs. Effective screening programs that combine tobacco cessation counseling, shared decision-making, and appropriate follow-up require the coordination of primary care providers, pulmonologists, radiologists, and frequently tobacco cessation counselors and are dependent on having a lung cancer screening coordinator ^9, 10^. Recent studies in the Veterans Affairs system have highlighted some challenges to the implementation of effective screening programs ^11^. There are now studies of the implementation of lung cancer screening which highlight the importance of having screening coordinators (also known as nodule navigators) to help manage patients over time ^10^. Though challenging, lung cancer screening in a high-risk population has the potential to reduce lung cancer mortality, and the recommendations need to be more routinely implemented in high-risk subjects.

## Lung cancer chemoprevention: interventions in pre-malignancy

Although detecting lung cancer at a stage when it is still curable will improve mortality, preventing the development of lung cancer would lead to large improvements in morbidity and mortality. Prior to the development of invasive carcinoma, there are distinct histologic pre-malignant lesions. Carcinogenesis for squamous cell carcinoma includes progression of pre-malignant lesions from squamous metaplasia to various levels of dysplasia, followed by carcinoma *in situ* and finally invasive squamous cell carcinoma. Bronchial dysplasia is an accepted intermediate for lung cancer chemoprevention trials. An analogous, though histologically distinct, process occurs in the development of adenocarcinoma, where the earliest lesions are called atypical adenomatous hyperplasia and adenocarcinoma *in situ* (these may present as ground-glass opacities on screening CT). Fortunately, not every pre-malignant lesion will progress to invasive carcinoma, although at this point it is difficult to predict which pre-malignant lesions will progress to invasive lung cancer.

Chemoprevention is the use of pharmacologic, or dietary, agents in order to prevent or slow the progression of cancer ^12^. Although the only proven approach to decrease the risk of lung cancer is smoking cessation, there have been multiple studies in the past, and a few ongoing, to identify effective chemopreventive agents for lung cancer.

Challenges in the lung cancer chemoprevention field have included identifying patients at high enough risk of developing lung cancer that using a preventive agent is warranted. Primary chemoprevention targets healthy individuals who are at increased risk, and for lung cancer this includes current and former smokers. Secondary chemoprevention focuses on preventing the progression of pre-malignant lesions into cancer. Tertiary chemoprevention refers to the prevention of a second primary tumor in patients who have had a previous lung or other tobacco-related aerodigestive cancer. With the new recommendations for lung cancer screening of high-risk individuals, there should be a shift to earlier-stage disease and improved survival. This will lead to more long-term survivors who remain at high risk for a second primary tumor and would be excellent candidates for chemoprevention. Given the “field cancerization” effect of tobacco smoke on the airway epithelium, there are frequently genetic changes to cells that have no gross or histologic abnormality, which could develop into dysplastic or malignant foci ^13, 14^. Collectively, there are significant opportunities for the identification of patients for primary, secondary, and tertiary chemoprevention.

Tobacco cessation is the bedrock of lung cancer prevention and currently is the only intervention that has been proven to reduce one’s risk of developing lung cancer. This has been demonstrated repeatedly; recently, data from the NLST showed that a sustained period of tobacco cessation decreased the risk of lung cancer death. After 7 years of abstinence from smoking, participants had a 20% reduction in lung cancer death, and this matches the risk reduction seen in those screened with low-dose CT. When both sustained smoking cessation and low-dose CT screening were used, there was a 38% risk reduction ^15^. Therefore, it is crucial that smoking cessation be part of lung cancer screening and of any lung cancer chemoprevention program ^9^.

For decades, researchers have been working to identify effective chemopreventive agents for lung cancer. Based on observational data which suggested benefit of supplements such as vitamins and minerals, multiple studies have been conducted which ultimately showed no benefit using multiple agents (summarized by Keith and Miller, 2013) or which even demonstrated harm with an increased risk of lung cancer (beta carotene) ^16^. These results highlight the importance of establishing safety and efficacy by using preclinical models.

The prostaglandin pathway is altered in a subset of subjects with squamous cell lung cancer. Building on preclinical evidence that this pathway may be important in lung cancer chemoprevention, a clinical trial of iloprost, an oral prostacyclin analog, demonstrated improved endobronchial histology, which is an accepted secondary endpoint for squamous cell lung cancer development. This improvement was seen in former smokers only, whereas current smokers in the trial did not show benefit and this again highlights the importance of tobacco cessation in lung cancer prevention. Although oral iloprost is not currently being produced, an active US clinical trial in lung cancer chemoprevention is evaluating the effect of inhaled iloprost on endobronchial histology (NCT02237183). The other trial uses aspirin in subjects with subsolid pulmonary nodules (NCT02169271) in a trial design that intersects lung cancer chemoprevention with screening and an investigation of potential biomarkers to predict response ^17^.

## The road forward: personalized screening and chemoprevention

With the recent changes in lung cancer screening, there is new momentum in the area of lung cancer pre-malignancy and early cancer. Although the field of cancer biology has been advanced by data sharing efforts such as The Cancer Genome Atlas, which contains sequencing information from thousands of cancer samples, the changes in pre-malignancy are less well understood. We support the proposal to create a Pre-cancer Genome Atlas, which would greatly improve the understanding of pre-malignant airway biology and help predict those lesions that ultimately will progress to invasive cancer. The ability to identify patients with pre-malignant lesions, and which lesions may progress to invasive carcinoma, would significantly improve the precision of monitoring and the early detection of lung cancer. This would intersect with chemoprevention as primary and secondary chemoprevention efforts could be applied in a more targeted, and therefore more effective, manner. Furthermore, as with invasive lung cancer, it is likely that not every individual, or every pre-malignant lesion, will respond to the same chemopreventive strategy. The ability to apply precision medicine methods to chemoprevention, as already occurs with lung cancer chemotherapy, should improve efficacy and focus clinical studies. As has been recently demonstrated, there is significant genetic heterogeneity, even within a single tumor, suggesting that intervening at a pre-malignant stage, when there is more homogeneity and fewer mutations, may prove both simpler and more effective ^18^. Immunotherapy has proven beneficial in lung cancer treatment, and harnessing the immune system to eradicate pre-malignant lesions is another future direction for clinical trials ^19– 22^.

At all stages of the disease, researchers and clinicians have an interest in using biomarkers from specimens that can be collected non-invasively (for example, plasma/serum, nasal or buccal brushings, exhaled breath, and urine). Screening biomarkers could identify patients at high risk of developing malignancy and may even help identify patients who could benefit from primary chemoprevention. Diagnostic biomarkers could help determine which patients with CT abnormalities have a malignancy, as opposed to benign pulmonary nodules. They could also help identify individuals who would benefit from secondary chemoprevention, as there are frequently ground-glass opacities on CT that are reflective of early stage adenocarcinoma. Prognostic biomarkers would provide important information about the behavior of a malignancy and could help target treatments.

There is a large body of research into biomarkers for these various stages, although most studies have focused on diagnostic and prognostic biomarkers using biospecimens, including serum, sputum, and even exhaled breath. A fundamental difficulty in lung cancer pre-malignancy is identifying which high-risk patients and lesions require intervention and which may respond to targeted chemoprevention. The use of accurate non-invasive biomarkers could revolutionize the current clinical approach. In terms of what can be used as a biomarker, circulating tumor cells (CTCs), circulating free tumor DNA (cfDNA), and microRNAs (miRNAs) are being evaluated in many lung cancer studies. CTCs and cfDNA have been shown to be specific, and their utility will undoubtedly increase as testing methods improve. The ability to detect malignant cells and to identify crucial mutations from non-invasively collected biospecimens would revolutionize the diagnosis and treatment of lung cancer ^18^. Although CTCs and cfDNA are being studied mostly in the setting of malignancy, biomarker studies increasingly are being incorporated into studies of early lung cancer and pre-malignancy, as in the current chemoprevention trials ^17^. For miRNAs, there are several in the literature that have been repeatedly identified as being relevant in tumors themselves, including miR-21, miR-31, miR-126, miR-143, miR-145, miR-182, miR-183, miR-200c, and miR-210 ^23, 24^. Unfortunately, consistently validating the same miRNAs in other specimens has been challenging, although their potential as biomarkers remains ^25– 29^. There are currently no non-invasive biomarkers validated for clinical practice for the diagnosis of early lung cancer or pre-malignancy.

While biomarkers remain in development, there are studies focused on making lung cancer screening a more individualized process. The NLST classified subjects as high risk on the basis of age, tobacco use, and years abstinent only. There are improved lung cancer risk calculators that include additional factors such as the presence of emphysema, a family history of lung cancer, and educational background ^30, 31^. There are patients who are at increased risk by these calculators but who would not have met the NLST criteria and who are not covered by the USPSTF recommendations; in fact, many patients who are diagnosed with lung cancer would not have met screening recommendations ^32^. Though more cumbersome to use, these risk calculators may identify additional high-risk patients who could benefit from lung cancer screening, although there is not always coverage by insurance for screening these patients.

There is an exciting convergence of opportunity in the fields of lung cancer chemoprevention and early detection—with the possibility of synergism. We hope that the field continues to pursue the best populations to screen, to characterize the highest-risk lesions, to identify effective chemopreventive agents, and to individualize the treatment of patients who can derive benefit.
